# Longitudinal Changes in the Physical Activity of Adolescents with Anorexia Nervosa and Their Influence on Body Composition and Leptin Serum Levels after Recovery

**DOI:** 10.1371/journal.pone.0078251

**Published:** 2013-10-21

**Authors:** Elzbieta Kostrzewa, Annemarie A. van Elburg, Nicole Sanders, Lot Sternheim, Roger A. H. Adan, Martien J. H. Kas

**Affiliations:** 1 Department of Translational Neuroscience, Brain Center Rudolf Magnus, University Medical Center Utrecht, University of Utrecht, Utrecht, The Netherlands; 2 Rintveld Centre for Eating Disorders, Altrecht Mental Health Institute, Zeist, The Netherlands; Monash University, Australia

## Abstract

**Objective:**

Patients with anorexia nervosa (AN) are often observed to have high levels of physical activity, which do not necessarily diminish after a successful therapy. Previous studies have shown that body fat tissue recovery in these patients is associated with a disproportional restoration of the adipocyte hormone, leptin. Therefore, we wondered whether the individual variation in physical activity in AN patients prior to treatment may be related to body fat percentage and plasma leptin level outcome.

**Method:**

Body fat percentage, leptin serum, and physical activity levels (accelerometer) were measured in adolescents with an (n=37, age 13 to 17.5 years) at initial assessment, at the end of study participation (median 12 months), and at one-year follow-up.

**Results:**

Accelerometer data were used to split the patients in two groups: those with low (n=26) and those with high levels of physical activity (HLPA, n=11). These groups did not differ in terms of age, IQ, presence of menses, BMI and season of admission. The HLPA group was characterized by a longer total duration of illness. Physical activity levels during therapy decreased for the group with initially HLPA and increased for the group with low levels of physical activity (to comparable levels). Physical activity remained stable after one year. The increase in body fat percentage and leptin levels were dependent on the recovery status; however, recovered patients with initially HLPA had significantly higher fat mass during the follow-up.

**Discussion:**

HLPA, an important modulator of AN progression in adolescents, can be successfully diminished by therapeutic intervention. Among recovered patients, those with initially HLPA had higher fat mass levels than those with low levels of physical activity. This finding suggests that HLPA are an important modulator of the body composition recovery mechanism.

## Introduction

Hyperactivity is one of the core symptoms of anorexia nervosa (AN)[1,2]. It was reported that about 30-80% of individuals with AN can be characterized as hyperactive [3]. Hyperactivity may be differentiated in AN patients in three ways: 1) excessive exercise (i.e. exercising at least 6 hours per week [4] or an exercise that, when postponed, evokes negative emotional states [5,6]); 2) high commitment to exercise despite its adverse consequences for ones health or social contacts [7]; or, 3) restlessness which may express itself in constantly active posture, fidgeting or inability to sit still [8,9]. Various studies have shown that hyperactive AN individuals differ from those who are not characterized by hyperactivity [10–12]. Hyperactive individuals with AN are at higher risk for relapse [10,11] and are usually diagnosed at a younger age [12]. It is suggested that AN sufferers exercise in order to deal with negative affections [13]. This may be reflected in studies that found positive [14] as well as a negative [15] correlations between levels of activity and anxiety measurements. Hyperactivity can manifest itself in various forms, such as restlessness and fidgeting or engagement in intensive physical exercise. Intensive physical exercise, though not pathological per se, facilitates body weight loss in the acute phase of the AN and hampers the efforts to restore body weight [12]. Just as high physical activity levels in AN patients may be a premorbid factor of the disease, the elevated physical activity levels assessed during an eating disorder (ED) may just reflect their premorbid high physical activity levels [4,16]. However, some argue that hyperactivity is a consequence of extreme food intake limitation [17,18]. Discrepancies were also observed in clinical evidence [13,16]. Because of these discrepancies, it is unclear whether one should expect a decrease in physical activity during treatment. To our knowledge, the issue of changes in physical activity levels during treatment and their consequences of these changes in recovery were never assessed in adolescents with ED. One exception is a case study reporting a slight but not complete decrease in hyperactivity in one adolescent ED patient after treatment [19].

Body fat percentage (fat%) drops severely in individuals with AN due to weight preoccupation, restricted feeding, and/or exercise regime. Patients’ interest is devoted to restricting fat intake with relatively maintained protein and vitamin intake [20]. As a consequence, percent body fat is even lower in persons with ED than in underweight females without ED matched for BMI [21–23]. Therapeutic interventions aim, among other goals, to restore body weight and body composition; however, various research groups have shown abnormalities in fat tissue restoration in AN patients after regaining body weight. Namely, fat tissue tends to restore faster in abdominal regions than at the extremities [24–29]. This effect is no longer observed one year after recovery [25]. 

Changes in body fat percentage at the acute phase of the disease [20] and during recovery [30] are reflected in the plasma levels of leptin, a hormone secreted by fat tissue. Leptin plays pleiotropic roles in energy assessment and expenditure. When animals are food deprived, and as a consequence develop hyperactivity, leptin injections diminish their activity level [31–33]. As a consequence, leptin levels were hypothesized to be inversely related to the heightened activity levels in ED. It has indeed been shown that high physical activity [34] and subjective motor restlessness [35] correlate with low plasma leptin levels. Weight restoration in individuals suffering from AN is followed by an increase in leptin, sometimes above the normal range [36–38]; however, hyperleptinemia is not always detected [39,40]. If hyperleptinemia occurs, leptin levels are said to drop back to a normal range within weeks or months [37]. The abrupt increase in leptin to levels exceeding those normally observed in age and BMI matched controls may hamper body weight restoration and was associated with heightened risk of relapse [14]. Hyperleptinemia can partly be a consequence of sudden increase in body fat percentage in weight-restored AN patients. Thus, body fat percentage changes can modulate changes in various ED symptoms, including hyperactivity and excessive exercise. Other hormonal changes were also observed in acutely ill individuals with AN, such as elevated levels of ghrelin [41,42], a hormone secreted by the stomach in response to fasting [43]. Body weight increase in recovered individuals with AN is accompanied by elevated ghrelin levels [41,44].

In the present study, we measured the physical activity of adolescent ED patients by using an accelerometer. The measurements were performed longitudinally: at the initial assessment, at the end of study (maximum of 12 months after the initial assessment) and at one-year follow-up. First, we tested the hypothesis that high levels of physical activity decrease in adolescent ED patients as a result of treatment. We also evaluated if this effect is still present at one-year follow-up. Second, we assessed whether high levels of physical activity at the acute phase of the illness (the initial assessment) would negatively influence the recovery rate. Finally, as body composition and leptin levels at recovery have an influence on relapse, we investigated if high levels of physical activity in AN patients influence the body composition and leptin levels restoration at recovery and at one-year follow-up. 

## Methods

### Participants

Female adolescent patients (n=51) were referred to the treatment center (by e.g., general practitioners or mental health professionals) because of an expected diagnosis of AN. Patients were between 13 and 17.5 years old and were still living with their parents. Six patients who did not fulfill the weight criterion, but whose weight was clearly below that which is expected from their own growth curves were diagnosed as having Eating Disorders Not Otherwise Specified and were also included in the study. Comorbidity was not common in our sample. The majority of patients (63%) were diagnosed with restrictive type of AN and the remaining with purging type of AN. The type of AN was not related to the physical activity level of the AN patients (Fisher exact test: P <0.44).

Study information included a letter sent to the patients’ home address followed by an interview with the subject, the parents, and a researcher during the first visit to the treatment center. Upon confirmation of the eating disorder diagnosis and after obtaining informed consent, a diagnostic assessment followed. All 51 patients were enrolled in a treatment procedure. Out of these, 14 patients were not included in the analysis due to missing data for objectively measured physical activity (PA) (in three cases, the Actiwatch malfunctioned; two patients did not wear the Actiwatch; and nine patients showed long periods of inactivity, which indicated Actiwatch misuse). Therefore, the final sample included in the current study consisted of 37 patients (23 inpatients and 14 outpatients). The sample was used in a previous study by Carrera and colleagues [15]; their study assessed the influence of ambient temperature on the expression of excessive physical activity in AN patients.

### Ethics statement

All participants provided a written informed consent. In case of minor participants, the written consent was also obtained from the next of kin. All procedures were approved by the Medical Ethics Committee of the University Medical Center Utrecht. 

### Treatment Center

The study took place between January 2006 and May 2009 at the Rintveld Center for Eating Disorders in Zeist (Altrecht Mental Health Institute). All treatments were covered by the Dutch health insurance and were without additional costs for patients and their families. Rintveld is a specialist center for eating disorders and is one of the two top clinical centers in The Netherlands. This center offers multidisciplinary in- and outpatient treatment for adolescent (<18 years) and adult patients. In addition, if necessary, medical treatment may be provided by the nearby Meander Hospital in Amersfoort. The staff of the adolescent treatment program consists of psychiatrists and other physicians, dietitians, family therapists, psychomotor therapists [45], creative therapists, psychologists, and nurses. An integrated approach that aimed at recovery of weight, eating pattern, and body attitude, as well as normalizing family relations and developmental and social skills, was offered to the patients in a system-oriented stepped and matched care model. Additional treatments were assigned to patients according to their needs and were adjusted during treatment as appropriate. Clinical staff aimed at limiting patients’ hyperactivity when possible. Patients attended school at home or at the center whenever possible. Weight gain was targeted at 0.5–1.0 kg/week in accordance with clinical guidelines.

### Time frame of the study

**Figure 1:**
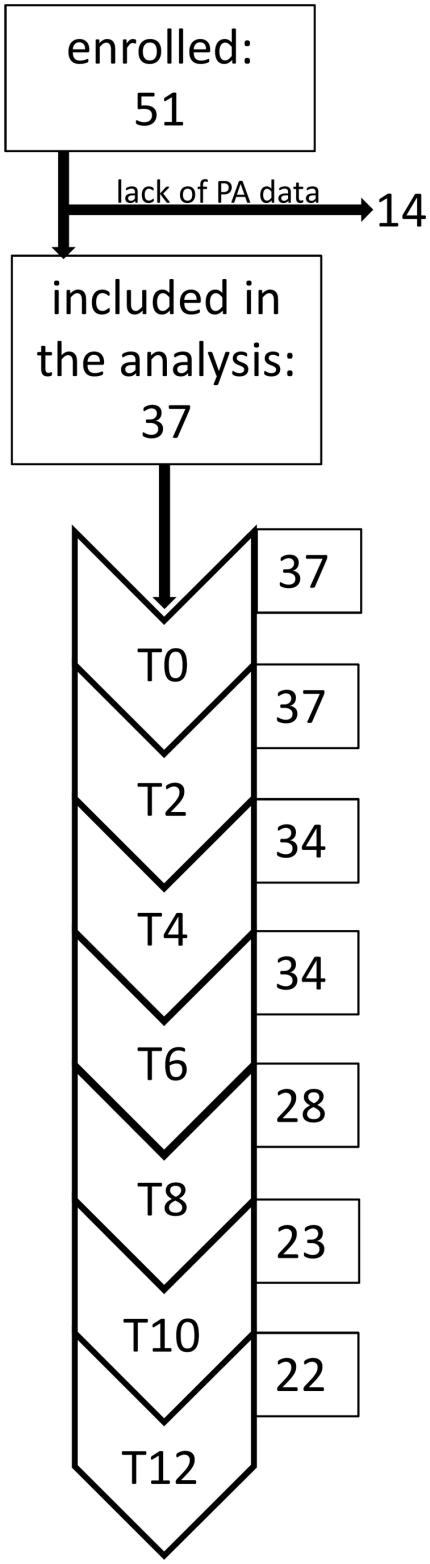
Timeframe of the study. The diagram shows the number of patients in the study at each data measurement point between enrolment, initial assessment (T0), and maximal duration of treatment (T12).

All patients obtained treatment for the full year or until they recovered or dropped out of the treatment. Additionally, patients took part in a longitudinal study with assessment repeated every second month for a maximum of one year. Thus, measurements were taken at initial assessment and at 2, 4, 6, 8, 10 and 12 months after the initial assessment. As most patients stayed in the treatment and participated in the study for the whole year, the median duration of the study was 12 months (minimum 2 months, maximum 12 months). Fifteen patients dropped out of the study sooner than 12 months (i.e., patients who withdrew between initial assessment and the 12th month, terminated treatment against advice, or terminated treatment with the agreement of their therapist after at least partial remission) (for overview, see Figure 1). The last assessment taken for each of the patients was considered “the end of study” for that patient. Measurements were taken also one year after the end of the study (follow-up). There was no difference in the duration of study participation between patients classified as having high levels of physical activity and those who were classified as having low levels of physical activity (Mann-Whitney test: U=129.00, *P* <0.66). Additionally, we calculated the duration of illness prior to the initial assessment (duration of illness) as a difference between age of AN onset and the date of admission to the clinic. 

### Data collection

#### At initial assessment

Demographic information collected during the first visit included the results of a physical examination, psychiatric interview, and an assessment of ED and comorbid psychopathology. A trained psychologist assessed the presence of AN characteristics based on DSM-IV criteria using the eating-disorder examination (EDE). Furthermore, we used standardized BMI (sdBMI) smaller than 1.0 (comparable with BMI 19 in adults) as the weight criterion for AN. Additionally, the patients were examined using the Eating Disorder Inventory-2 (EDI-2) (Garner 1991); the Morgan and Russell Outcome Assessment Schedule (MROAS); the Comprehensive Psychopathological Rating Scale (CPRS-S-A); and the Information, Vocabulary, Block Design, and Picture Completion tasks of the Wechsler Intelligence Scale for Children-3 (WISC3). Finally, physical activity, fat%, as well as serum leptin and ghrelin levels, were measured.

#### At the end of study and at follow-up

Data on sdBMI, presence of menses, EDI-2, MROAS, CPRS-S-A, physical activity, fat%, serum leptin, and ghrelin levels were collected at the end of study and at follow-up. 

### Recovery definition

For study purposes, we used MROAS to split the patient population into the recovered (MROAS score ≥ 9) and the non-recovered group (MROAS score < 9). Patients were classified as recovered or non-recovered at the end of study and at follow-up study independently. The MROAS is often used in AN research and provides a quantitative measure (scores 0–12) of current outcome state divided over five subscales: nutritional state, menstrual function, mental state, psychosexual adjustment, and socio-economic status. Higher MROAS scores indicate better outcome states. The composite MROAS score can also be used to evaluate patient’s outcome state in three categories: Good (composite score ≥9), Intermediate (≥4 and ≤8), and Poor (<4) [46].

### Standardized BMI (sdBMI)

We used BMI transformed into sdBMI scores on the basis of the standard growth curves for the Dutch population (Growth Analyser 3.5, Dutch Growth Foundation, Rotterdam, NL). The use of the sdBMI values instead of BMI values was motivated by the fact that this study involved adolescent patients (13.5 - 17 years of age) and because we analyzed the longitudinal data of each patient (BMI at intake and at the end of study). Average BMI changes significantly during puberty. It may reach values considered to be low according to standard cut-off points. However, these values may still be within a healthy range when the standard growth curves for the age and gender are considered. Adolescent patients should gain weight at a level that is considered appropriate for their age. The amount of body weight loss during illness is potentiated by the fact that they drift away from their normal growth curve. Therefore, sdBMI, in comparison with BMI, is considered a more precise description of the body growth of adolescents.

### Objective assessment of physical activity

Physical activity (PA) was measured using an accelerometer (Actiwatch model AW 4; Cambridge Neurotechnology, Cambridge, United Kingdom). The Actiwatch was strapped to the patient’s right ankle and was worn for three consecutive weekdays, from 9 pm on the first day to 9 pm on the fourth day, except while swimming and showering. The epoch length (sampling time) for the Actiwatch was set to 1 minute. Night activity (23:00–07:00) and sequences of >10 min of consecutive zero counts were excluded from the recordings. This procedure was similar to that recently used in the field of eating disorders [13]. Thereafter, the data were summarized as counts per day and patients were excluded from analyses if more than 30% of the day was not available for two of the three days. Activity data from days 1 to 3 were averaged to determine daily physical activity for each patient. Data analysis was undertaken to determine the periods of time (%) at varying intensity levels of physical activity. The ranges (in counts per minute) for the activity intensities were <200 for sedentary activity (SA), 200 to <1800 for light activity (LA), and ≥1800 for moderate-to-vigorous physical activity (MVPA), as validated by Puyau et al. [47] for the Actiwatch device worn on the lower right leg. Patients were subdivided into groups characterized by high levels of physical activity and low levels of physical activity (HLPA and LLPA groups, respectively) on the basis of their participation in moderate or vigorous physical activity measured by the accelerometer. Accelerometer calibration studies showed that MVPA readouts correspond to high levels of physical activity such as participation in sports [47]. Thus, total time spent on MVPA was used to split the population into the two groups by using a cutoff of 2.5 hours of MVPA readout per three days of measurements, which corresponds to the definition used in previous publications [13]. 

### Physiological parameters

Body weight and fat% were measured using a TANITA Body Composition Analyzer TBF-300 (Tanita Corporation, Tokyo, Japan). Blood samples were obtained by venipuncture for hormonal analysis. Plasma samples were stored at −80°C prior to determination. Leptin was measured using a sensitive Radio-Immuno-Assay (RIA) (Sensitive Human Leptin RIA Kit, Linco, St. Charles, Missouri, U.S.A.), intra-assay CV of 5.63%, and inter-assay CV of 5.66%. Ghrelin was measured using a sensitive Radio-Immuno-Assay (RIA) (Human (total) Ghrelin RIA Kit, Linco, St. Charles, Missouri, U.S.A.), intra-assay CV of 6.4%, and inter-assay CV of 16.3%. Relative hyperleptinemia was defined as leptin levels above the 95 percentile for a given BMI for girls 5.8-18.99 years old based on previously published observations [37].

### Data analysis

Data of all patients with valid data for at least the first two months of the study were analyzed. Missing data were imputed according to the last-observation-carried-forward method. According to this method, the last observed data point after initial assessment may be used for all subsequent missing data points until the end of the study for a given patient. Student’s t-test was used to compare the LLPA and the HLPA groups as well as the recovered and the non-recovered groups. Effect size for the t-test results was calculated using Pearson’s r. Repeated measures ANOVA (with Greenhouse-Geisser correction if necessary; in that case ɛ value is reported) was used to analyze physical activity data at initial assessment, at the end of study and at follow-up. Effect size for repeated measures ANOVA was calculated using partial Eta^2^ (η^2^_partial_). Fisher exact test was used for the in- and outpatient analysis. Chi-square and Kaplan-Meier analyses were used to evaluate recovery and recovery rate differences between the LLPA and the HLPA group. Cramer’s V coefficient was used as an estimate of the effect size for the chi-square test. Two-way ANOVA, with physical activity group classification and recovery status as main factors, was used to compare the characteristics of the recovered and the non-recovered LLPA and HLPA patients. In all cases, Levene’s test of equality of error variances showed that the assumption of the two-way ANOVA was met. Spearman’s rho correlation analysis was used to examine associations between fat%, plasma leptin, and plasma ghrelin levels with psychological variables measured with EDI-2 and CPRS-S-A. All analyses were performed with SPSS 20.0 (IBM) and data are presented as mean ± standard error of the mean. For significance thresholds, a *P*-value of 0.05 was used throughout, unless the *P*-value was corrected for multiple comparisons as stated at the results section.

## Results

### Population characteristics

All the participants (n=37, all those who had physical activity data at initial assessment) were females, aged 13 to 17.5 years at initial assessment (mean=15.15, SD=1.21), with average IQ of 104.8 (SD=13.11), and BMI between 12.6 and 18.4 (mean=15.66, SD=1.38). Out of those patients, 30% were classified as belonging to the HLPA group at initial assessment based on the objective accelerometer data (LLPA n=26, HLPA n=11). 

### Recovery

At the end of study, 51.4 % of patients met the criteria for recovery according to MROAS (19 recovered (Rec), 18 non-recovered (nonRec) patients). The Rec group gained more body weight than the nonRec group; however, the two groups did not differ significantly in terms of BMI at the end of study. At this time point, the Rec and nonRec group differed significantly in terms of the Obsession and Depression scales of CPRS-S-A, as well as for Maturity Fears and Social Insecurity scales of EDI-2 (Table 1). These differences were observed despite the fact that at initial assessment, there were no differences between the Rec group and the nonRec group for CPRS-S-A or EDI-2 (data not shown). Between the end of study and the follow-up, six additional patients recovered. However, six other patients relapsed. It is of note that the relapse rate in our sample (16%) was comparable to the one reported in the literature [11]. At one-year follow-up, the nonRec group had a significantly lower BMI and sdBMI than the Rec group (Table 2). Furthermore, the nonRec group had higher scores than the Rec group for CPRS-S-A (all scales) as well as for EDI-2 (Ineffectiveness, Interpersonal Distrust, Interoceptive Awareness, Maturity Fears, and Social Insecurity) (see Table 2).

**Table 1:** Characteristics of the sample at the end of study.

	**nonRec (n=18)**	**Rec (n=19)**			
	**Mean (SD)**	**Mean (SD)**	**t(df)**	***P<***	**e.s.**
**BMI**	18.656 (0.424)	19.311 (0.345)	-1.205 (35)	0.24	0.200
**sdBMI**	-1.033 (0.260)	-0.479 (0.189)	-1.73 (31.4)	0.09	0.295
**CPRS-S-A**					
**anxiety**	6.563 (4.60)	5.250 (3.553)	0.937 (32)	0.36	0.163
**obsessions**	8.781 (5.709)	4.056 (3.753)	2.81 (25.4)	**0.01**	0.487
**depression**	8.562 (6.129)	4.722 (4.226)	2.147 (32)	**0.04**	0.355
**EDI-2**					
**drive for thinness**	33.28 (7.61)	27.89 (10.48)	1.780 (35)	0.08	0.288
**body dissatisfaction**	41.94 (10.50)	36.47 (12.47)	1.439 (35)	0.16	0.236
**bulimia scale**	13.11 (3.71)	11.53 (4.85)	1.112 (35)	0.27	0.185
**ineffectiveness**	37.06 (9.69)	33.89 (8.35)	1.065 (35)	0.29	0.177
**perfectionism**	19.61 (5.71)	18.11 (4.98)	0.856 (35)	0.40	0.143
**interpers. distrust**	22.89 (6.22)	21.05 (5.17)	0.979 (35)	0.33	0.163
**interoceptive aw.**	29.78 (8.34)	30.16 (7.53)	-0.146 (35)	0.86	0.025
**maturity fears**	27.11 (7.50)	22.79 (5.25)	2.041 (35)	**0.05**	0.326
**ascetism**	28.50 (7.72)	25.53 (6.30)	1.287 (35)	0.21	0.213
**impulse regulation**	27.33 (5.81)	25.16 (6.34)	1.086 (35)	0.29	0.181
**social insecurity**	30.33 (6.91)	25.95 (5.98)	2.068 (35)	**0.05**	0.330

Characteristics of the nonRec (non-recovered) and the Rec (recovered) groups at the end of study. EDI-2 = Eating Disorder Inventory, Interpers. distrust = interpersonal distrust; interoceptive aw.= interoceptive awareness, CPRS-S-A = Comprehensive Psychopathological Rating Scale, e.s. = effect size. Student’s t-test, significant results are in bold.

**Table 2:** Characteristics of the sample at follow-up.

	**nonRec (n=18)**	**Rec (n=19)**			
	**Mean (SD)**	**Mean (SD)**	**t(df)**	***P* <**	**e.s.**
**BMI**	17.95 (1.95)	19.71 (1.72)	-2.918 (35)	**.006**	0.442
**sdBMI**	-1.66 (1.35)	-0.47 (0.71)	-3.374 (35)	**.002**	0.495
**CPRS-S-A**					
**anxiety**	8.50 (5.26)	4.36 (3.58)	2.711 (32)	**0.01**	0.432
**obsessions**	9.47 (5.43)	4.47 (3.82)	3.129 (32)	**0.01**	0.484
**depression**	9.59 (5.38)	4.44 (4.36)	3.077 (32)	**0.01**	0.478
**EDI-2**					
**drive for thinness**	31.78 (6.49)	28.11 (12.17)	1.15 (27.7)	0.26	0.213
**body dissatisfaction**	39.78 (8.39)	39.32 (12.50)	0.13 (31.6)	0.89	0.023
**bulimia scale**	14.61 (5.82)	11.58 (3.67)	1.906 (35)	0.07	0.307
**ineffectiveness**	38.56 (9.61)	31.47 (10.00)	2.194 (35)	**0.04**	0.348
**perfectionism**	19.39 (4.63)	16.42 (4.86)	1.901 (35)	0.07	0.306
**interpers. distrust**	24.33 (6.70)	19.58 (5.32)	3.397 (35)	**0.02**	0.498
**interoceptive aw.**	31.72 (7.34)	26.47 (7.35)	2.172 (35)	**0.04**	0.345
**maturity fears**	27.11 (6.86)	22.89 (5.04)	2.139 (35)	**0.04**	0.340
**ascetism**	27.11 (7.38)	23.11 (7.09)	1.684 (35)	0.10	0.274
**impulse regulation**	28.17 (6.53)	24.11 (7.04)	1.817 (35)	0.08	0.294
**social insecurity**	31.33 (6.08)	23.74 (5.38)	4.030 (35)	**0.01**	0.563

Characteristics of the nonRec (non-recovered) and the Rec (recovered) groups at follow-up. EDI-2 = Eating Disorder Inventory, Interpers. distrust = interpersonal distrust; interoceptive aw.= interoceptive awareness, CPRS-S-A = Comprehensive Psychopathological Rating Scale, e.s. = effect size. Student’s t-test, significant results are in bold.

### Characterization of LLPA and HLPA groups

**Figure 2:**
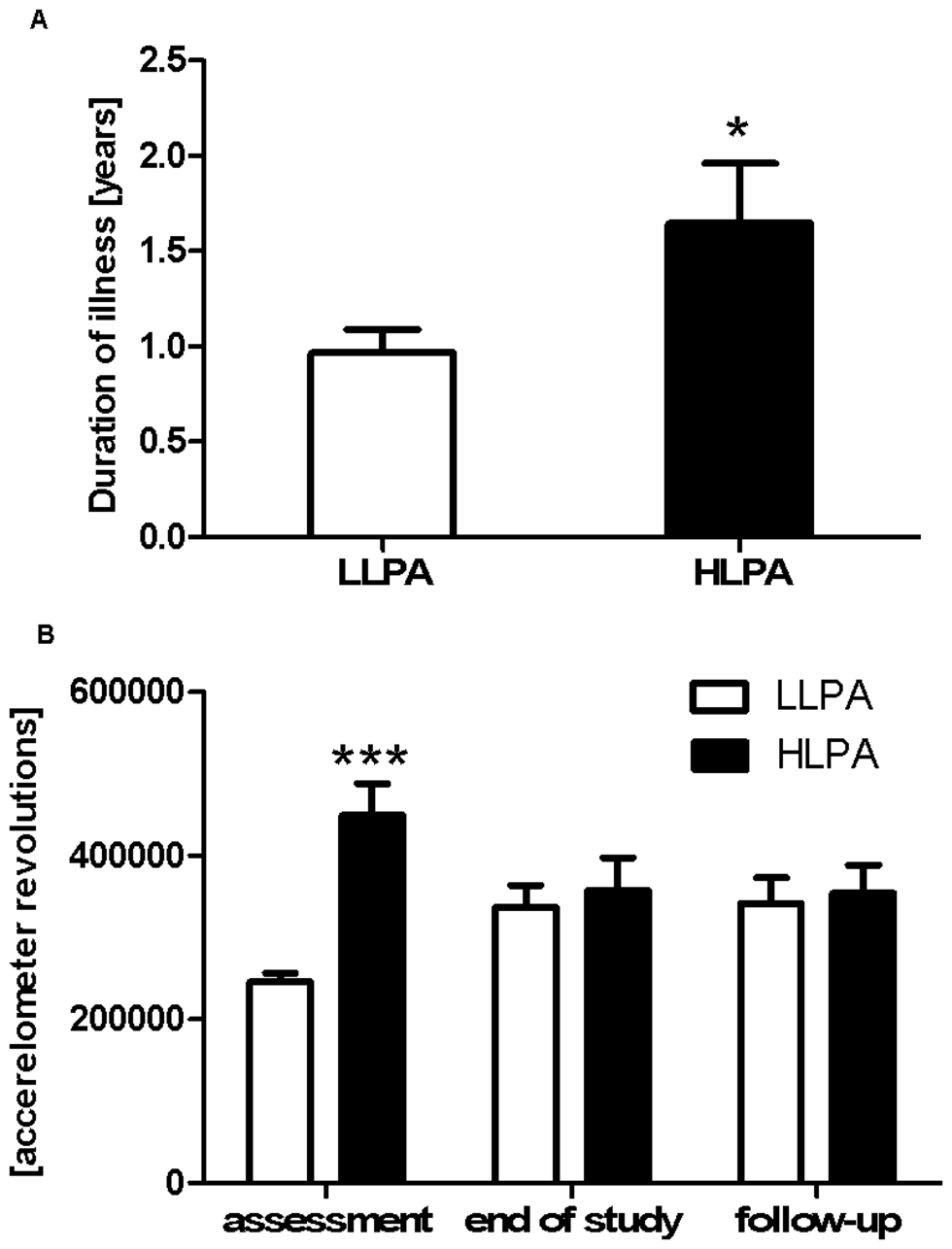
Characterization of LLPA and HLPA patients. A) Duration of illness, defined as time between obtaining the first diagnosis and initial assessment at Rintveld clinic. Duration of illness is significantly higher for the HLPA (high levels of physical activity) group than for the LLPA (low levels of physical activity) group. Mean±SEM, Student’s t-test, * *P*<.05. B) Physical activity of the LLPA and the HLPA groups at initial assessment, at the end of study, and at follow-up. Data are expressed as mean ± SEM; Repeated measures ANOVA, Student’s t-test as a post hoc test: *** *P*<.001 in comparison to the LLPA group at the same time point.

We compared the LLPA and HLPA patients at initial assessment to test whether other characteristics co-occurred in our sample of patients with high levels of physical activity. No differences between the LLPA and the HLPA groups were observed for age, IQ (n_(nonEE)_=23, n_(EE)_=11), BMI, presence of menses (n=31 no menses, n=1 regular menses, n=4 premenarche, n=1 use of anticonceptive pill), EDI-2 total score, EDI-2 subscales, and CPRS-S-A subscales (Table 3). Furthermore, outpatients were not more likely to be included in the HLPA group than inpatients (LLPA inpatients (n=15), LLPA outpatients (n=11), HLPA inpatients (n=8), HLPA outpatients (3); Fisher’s exact test, *P*<0.48). However, the LLPA and the HLPA patients differed in terms of duration of illness prior to initial assessment (Figure 1). Student’s t-test showed that the HLPA group had significantly longer duration of illness before admission to Rintveld Clinic than the LLPA group (t(35)=-2.472, *P*<0.02, r=0.99) with a large effect size (Figure 2A). This data indicate that the HLPA group had an earlier age of onset of the illness. 

**Table 3:** Basic characteristics of the sample split by physical activity levels.

	**LLPA (n=26)**	**HLPA (n=11)**			
	**Mean (SD)**	**Mean (SD)**	**t(df)**	***P*<**	**e.s.**
**Age**	15.262 (1.353)	15.345 (1.010)	-0.184 (35)	0.86	0.031
**IQ**	103.783 (12.446)	106.755 (13.124)	-0.640 (32)	0.53	0.112
**BMI**	16.188 (1.408)	15.582 (1.654)	1.138 (35)	0.26	0.189
**sdBMI**	-2.185 (1.026)	-2.827 (1.755)	1.398 (35)	0.17	0.229
**CPRS-S-A**					
**anxiety**	9.340 (4.377)	11.556 (4.572)	-1.287 (32)	0.21	0.222
**obsessions**	9.280 (4.880)	12.833 (5.006)	-1.861 (32)	0.07	0.313
**depression**	9.827 (13.222)	13.222 (5.197)	-1.657 (33)	0.11	0.277
**EDI-2**					
**drive for thinness**	34.115 (8.155)	35.500 (5.418)	-0.515 (35)	0.61	0.087
**body dissatisfaction**	42.269 (10.267)	42.818 (10.255)	-0.149 (35)	0.88	0.025
**bulimia scale**	12.962 (5.466)	15.182 (6.493)	-1.068 (35)	0.29	0.178
**ineffectiveness**	38.31 (8.57)	42.77 (8.29)	-1.462 (35)	0.15	0.240
**perfectionism**	19.04 (5.80)	20.27 (5.12)	-0.611 (35)	0.55	0.103
**interpers. distrust**	22.50 (5.94)	26.09 (5.75)	-1.696 (35)	0.10	0.276
**interoceptive aw.**	34.19 (7.80)	34.82 (6.95)	-0.230 (35)	0.82	0.039
**maturity fears**	26.54 (5.94)	30.41 (7.79)	-1.650 (35)	0.11	0.269
**ascetism**	27.92 (5.95)	30.10 (6.59)	-0.955 (34)	0.35	0.162
**impulse regulation**	27.43 (7.55)	29.40 (6.87)	-0.720 (34)	0.48	0.123
**social insecurity**	28.23 (4.94)	31.00 (6.73)	-1.360 (34)	0.18	0.227

Student’s t-test showed no differences between the LLPA (low levels of physical activity) and the HLPA (high levels of physical activity) groups at initial assessment. EDI-2 = Eating Disorder Inventory, Interpers. distrust = interpersonal distrust; interoceptive aw.= interoceptive awareness, CPRS-S-A = Comprehensive Psychopathological Rating Scale. All effect sizes (e.s.) are small or negligible.

### Decrease in physical activity during treatment

In order to test the hypothesis that physical activity decreases during treatment, we compared the time course of total physical activity between LLPA and HLPA groups at initial assessment, at the end of study, and at one-year follow-up. We conducted repeated measures ANOVA to assess changes over time of the physical activity levels of the patients. The physical activity levels were measured using an accelerometer and the patients were grouped on the basis of their physical activity (PA) levels. For daily physical activity levels, both the PA classification and the interaction between time and PA classification were significant, thus indicating that PA levels differed between the LLPA and the HLPA groups in a time-dependent manner. The Student’s t-test, which was conducted as a post hoc test (corrected *P*-value: p=0.0167), showed that the HLPA group had a significantly higher total daily physical activity than the LLPA group at initial assessment (t(38.37)=-9.137, *P*<0.001, r=0.99) (Figure 2B and Table S1). This difference disappeared during treatment due to the fact that the HLPA group decreased in their total activity while the LLPA group on average slightly increased in their activity.

### Influence of HLPA on recovery rate

**Figure 3:**
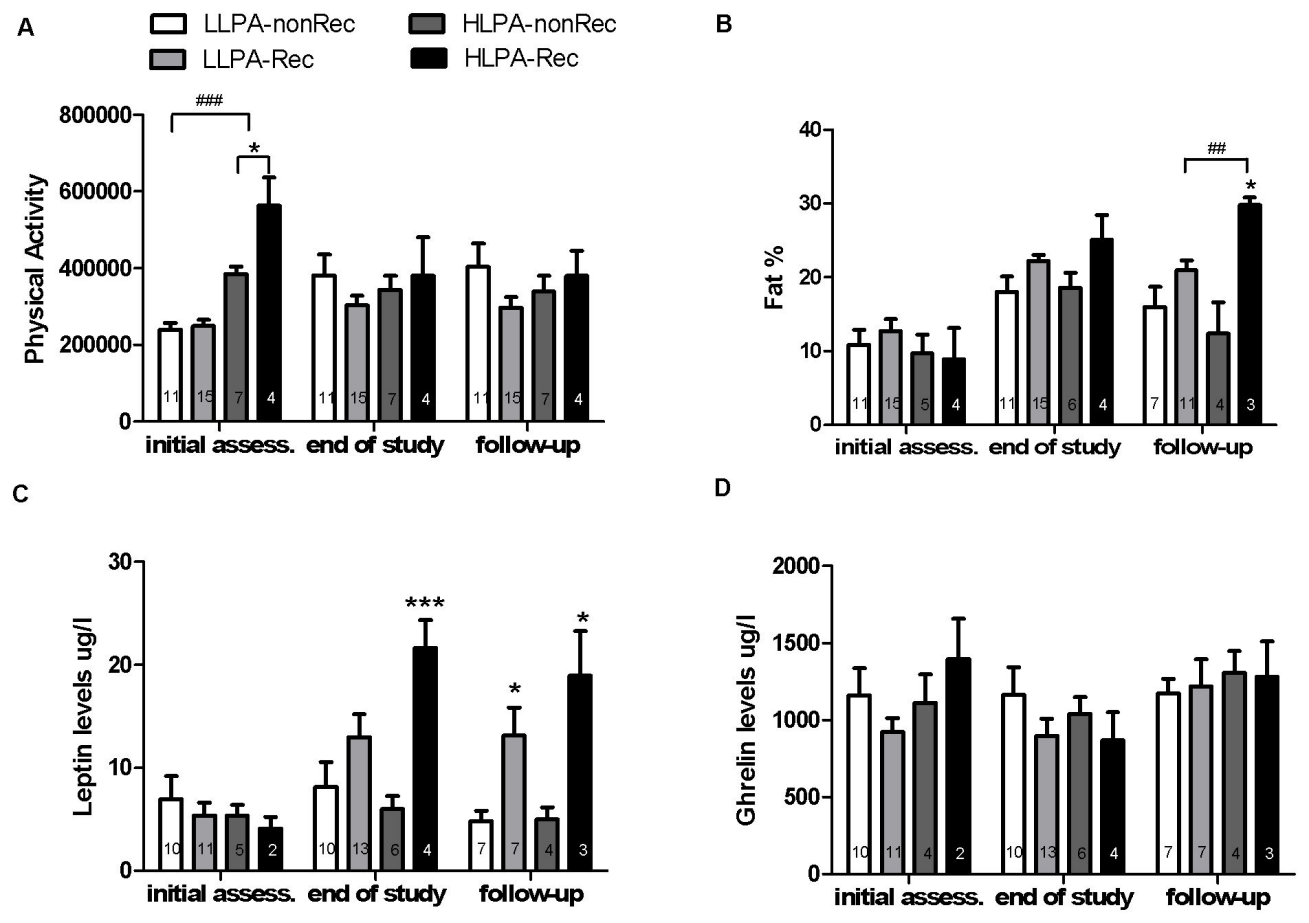
Differences between recovered and non-recovered LLPA and HLPA patients. Daily physical activity scores (A), fat% (B), leptin serum levels (C), and ghrelin serum levels (D) plotted for recovered and non-recovered LLPA and HLPA groups at 3 time points (initial assess. = initial assessment). Data are expressed as mean ± SEM; Two-way ANOVA, Student’s t-test as a post hoc test: * *P*<.05, *** *P*<.001 between the recovered (Rec) and non-recovered (nonRec) patients in the same group (LLPA or HLPA) and at the same time point; ## *P*<.01, ### *P*<.001 between the LLPA and the HLPA groups at the same time point. Numbers within bars indicate sample size per group.

We aimed at assessing if HLPA detected at initial assessment diminished the recovery rate of adolescent AN patients. There was no difference in recovery rate for both the LLPA and the HLPA groups (χ^2^(1)=1.408, *P*<0.24, Cramer’s V=0.195, for sample sizes see Figure 3A). Kaplan-Meier survival analysis with PA classification as a factor showed that there was no effect of PA classification on the recovery rate (mean=10.72, 95% CI 9.57-11.87; mean=10.57, 95% CI 8.87-12.28, for the LLPA and the HLPA groups respectively). Thus, our data suggested that HLPA at initial assessment had no negative influence on recovery rate of the adolescent AN patients. 

### Changes in PA levels in recovered and non-recovered patients

We tested the hypothesis that HLPA patients who recovered had lower physical activity levels at initial assessment than those who did not recover. We also assessed if changes in levels of PA were more profound for the Rec than for the nonRec patients from the HLPA and the LLPA groups. In order to do so, we split the population into four groups on the basis of PA classification and of recovery according to MROAS. As a consequence four groups were compared: LLPA-nonRec, LLPA-Rec, HLPA-nonRec, and HLPA-Rec. We conducted two-way ANOVA, with PA classification and recovery status as factors, to assess daily physical activity levels at assessment, at the end of study, and at follow-up (Figure 3A, Table S2). 

At the initial assessment, there was a significant effect of PA classification, recovery status, and interaction on daily physical activity levels. Post hoc analysis (Student’s t-test, corrected *P*-value: p=0.025) showed significant difference between Rec and nonRec patients in the HLPA group with a large effect size (t(9)=-2.989, *P*<0.02, r=0.71). HLPA patients, who recovered at the end of study, had significantly higher physical activity levels at initial assessment than those patients who did not recover. At the end of the study and at follow-up, the effect of PA classification, recovery status, and interaction between the two variables were not significant.

### Influence of HLPA on restoration of fat% and on plasma leptin levels

We investigated the influence of HLPA at initial assessment on the restoration of fat% and on plasma leptin and ghrelin levels after treatment (at the end of study and at one-year follow-up). For that purpose, we split the population into four groups as explained in the analysis above. We conducted a two-way ANOVA analysis, with PA classification and recovery status as factors, to characterize fat% at initial assessment, at the end of study and at follow-up. Due to the availability of a small number of blood samples for patients in the HLPA-Rec group at initial assessment, we only analyzed leptin and ghrelin levels at the end of study and at follow-up. 

Results for fat% are shown in Figure 3B and Table S2. At initial assessment, there were no differences in terms of fat% between the recovered and the non-recovered HLPA and LLPA groups. At the end of study, Rec patients had higher fat% than nonRec patients and there was no significant effect of PA classification or interaction on body fat%. At follow-up, recovered patients had higher fat% than non-recovered patients and this effect depended on PA classification as the interaction effect was significant. Post hoc analysis (Student’s t-test; corrected *P*-value: p=0.025) showed significant difference between the LLPA and the HLPA recovered patients with a large effect size (t(12)=-3.309, *P*<0.007, r=0.99). There was also a significant difference, with a large effect size, between recovered and non-recovered HLPA patients (t(5)=-3.424, *P*<0.02, r=0.69); however, there was no difference between recovered and non-recovered LLPA patients (t(16)=-1.828, *P*<0.09, r=0.42). 

Results for leptin levels are shown in Figure 3C and Table S2. Two-way ANOVA showed that at the end of study, the recovery status had a significant large effect on plasma leptin levels; however the PA classification or interaction had no effect. Post hoc analysis (Student’s t-test; corrected *P*-value: p=0.025) showed significant difference between recovered and non-recovered HLPA patients for leptin levels with a large effect size (t(8)=-5.886, *P*<0.001, r=0.90). Also at the follow-up, there was a significant effect of recovery status on leptin levels but not significant effects of PA classification or interaction. The effect sizes for PA classification and interaction were large, suggesting that a larger sample size might be required to obtain significant effect. Post hoc analysis (Student’s t-test; corrected *P*-value: p=0.025) showed significant differences (with large effect sizes) between recovered and non-recovered patients in the LLPA group (t(12)=-2.859, *P*<0.02, r=0.64) and the HLPA group (t(5)=-3.605, *P*<0.02, r=0.85). Results for ghrelin levels are shown in Figure 3D and Table S2. There were no significant effects for ghrelin at the end of study and at follow-up.

### Associations of psychological measurements with observed biological parameters

We assessed how the biological measures relate to the psychological measures at one-year follow-up in our sample. We used Spearman’s rho correlation analysis to examine associations between fat%, plasma leptin, and plasma ghrelin levels with psychological variables measured with EDI-2 and CPRS-S-A (Table S3). Analysis showed that fat% at follow-up correlated significantly and negatively with all the subscales of CPRS-S-A and with four EDI-2 subscales (Ineffectiveness, Interpersonal Distrust, Maturity Fears and Social Insecurity). As fat% and leptin correlated significantly (Spearman’s rho = 0.569, *P*<0.01), it is not surprising that plasma leptin levels at follow-up correlated significantly and negatively with all the subscales of CPRS-S-A and with six EDI-2 subscales (Ineffectiveness, Interpersonal Distrust, Interoceptive Awareness, Maturity Fears, Impulse Regulation, and Social Insecurity). Finally, ghrelin plasma levels correlated significantly and positively with two EDI-2 subscales (Body Dissatisfaction and Interpersonal Distrust). Taking under consideration the number of correlations, 5% of the significant associations are false positives (about 3 of the 42 correlations). Therefore, majority of the correlations obtained should be considered valid.

## Discussion

We conducted a longitudinal study in adolescent anorexia nervosa patients to assess the effects of treatment on high physical activity levels as well as to investigate the consequences of high levels of physical activity on physiological recovery. We showed that patients who had high levels of physical activity at initial assessment were characterized by earlier age of onset of the illness. During the treatment, physical activity levels of AN patients normalized (decreased for the HLPA and increased for the LLPA groups) at the end of treatment and remained stable at one-year follow-up. High levels of physical activity at initial assessment did not influence recovery rate in this sample. However, patients who engaged in high levels of physical activity had profound increases in fat% and leptin levels at recovery. The changes in body composition and leptin levels observed at recovery were still maintained at one-year follow-up.

The data showed the normalization of physical activity levels of the adolescent AN patients during treatment, and this effect was maintained one year later. Patients who were initially highly active diminished their physical activity, while patients who were characterized by low activity increased their physical activity. The increase in the PA of patients from the LLPA group may be potentially explained by the emaciation of patients at initial assessment, which might have resulted in decreased physical activity levels. As patients from the LLPA group partly restored their body fat%, their physical activity levels increased. As far as the decrease of physical activity of patients in the HLPA group is concerned, there are two possible explanations for this finding. First, this effect may be considered a positive verification of the hypothesis that extreme food limitation and body weight loss may be evolutionarily conserved triggers for hyperactivity observed in AN patients [14,17]. Second, the decrease in activity at the end of study might have been caused by the efforts of the clinical staff to decrease patients’ hyperactivity. The effect of reduction of PA was present also at follow-up. This may suggest that members of the patients' social surroundings learned about the negative effect of hyperactivity on the health status of AN patients. As a result, they may put more pressure on patients to stay less active. Finally, we cannot fully exclude the possibility that the observed normalization of physical activity seen in both groups is merely an effect of regression toward the mean. The latter would take place if the PA scores of both the LLPA and the HLPA groups at initial assessment were extreme in comparison to the average scores of the adolescent AN population. However, this is not the case. In a new study that is currently being conducted at the Rintveld clinic, a new group of adolescent AN patients has comparable PA scores to the population used in the current study. Also, the percentage of patients classified as HLPA is identical between the two populations. 

Contrary to what is expected, our data show that HLPA patients who recovered at the end of study were characterized by even higher physical activity levels at initial assessment than the non-recovered HLPA patients. The data may support previous findings that high levels of physical activity do not necessarily result in poorer outcomes after treatment. It was previously shown that, in AN patients, high physical activity levels are partly driven by body weight preoccupation [48]. Furthermore, the relation between high exercise levels and disordered eating is partially mediated by reasons to exercise [49]. These variables were, however, not assessed in the current study.

Previously, it was reported [12] that AN patients who were highly physically active were younger at the first clinical interview. Our findings also showed that the age of onset of AN was earlier in the HLPA group than in the LLPA group. We propose that high levels of physical activity may mask the progress and severity of AN for patients and their social surroundings. This is in agreement with previous hypotheses that one of the functions of high physical activity in individuals with AN is the denial of the negative outcomes of severe dieting [16].

The recovery rate during one-year follow-up was comparable to those previously reported in the literature [50]. As was predicted, recovery was associated with an increase in body fat% and leptin levels. In our sample, the fat% increase was more profound in a group of patients who were classified to the HLPA group at the acute phase of the illness. The effect on body fat% was maintained and even strengthened at one-year follow-up. Thus, we suggest that previous history of HLPA changes fat pad restoration in response to re-feeding. We also observed relative hyperleptinemia (assessed dependent on the BMI for age and gender matched controls) during recovery, consistent with previous reports [36,37]. However, contrary to a previous report [37], elevated leptin levels did not normalize at one-year follow-up. Although, 1) leptin levels at the end of study and at follow-up mimicked the group differences observed in body fat% at these time points; and, 2) though relative hyperleptinemia (assessed dependent on the BMI for age and gender matched controls) was observed in all of the HLPA-Rec patients and only in half of the LLPA-Rec patients, the results were not statistically significant. This is most probably due to the small HLPA-Rec group size and high variability in leptin levels. Finally, both fat% and leptin plasma levels in the current study correlated negatively at follow-up with psychological measurements such as anxiety, obsessions, depression (as measured with CPRS-S-A), and various subscales of EDI-2. These findings suggest that the higher fat% and leptin levels are at one-year recovery, the better the psychological outcome as measured by CPRS-S-A and EDI-2 (lower scores of psychopathology measured by these questionnaires). 

Although plasma leptin levels correlate with body fat%, leptin levels are likely to respond to short-term changes due to various stimuli such as food intake. As a consequence, slow changes in fat% are more informative of patients’ physical state than the dynamic leptin measurements. Nevertheless, plasma leptin levels may convey important information. First, in the current study, at follow-up, leptin was a much better predictor of psychological outcome than fat% measurement. Second, leptin levels may inform us about the propensity of a person to develop hyperactivity. Gelegen et al. in 2007 [51] showed that a mouse strain that is sensitive to the activity-based anorexia model has higher baseline plasma leptin levels in comparison to a strain that is more resistant to activity-based anorexia. Both strains are characterized by comparable plasma leptin levels as a consequence of restricted feeding. Thus the susceptible mouse strain (that becomes hyperactive during food restriction) is characterized by a stronger decline of leptin between baseline and experimentally evoked food restriction. This could lead to the hypothesis that the stronger the decline of leptin due to food restriction, the higher the sensitivity is to restricted caloric assessment and perhaps to the hyperactivity that is evoked by it. Additional research is needed to further test this hypothesis. 

We did not observe any effects of recovery on ghrelin levels. The high within-group variance of ghrelin levels suggests that factors, other than the ones included in the current study, may have influenced ghrelin levels.

The main limitation of the study is the drop-out of patients between initial assessment, end of study, and follow-up. As a consequence, some of the groups have a small headcount. Therefore, it is not justified to generalize the findings on the influence of HLPA on recovery of fat% and leptin levels to all AN patients. Another limitation of the study is the lack of a control group, which makes it impossible to state whether the HLPA group is characterized with higher physical activity levels than healthy controls or only relative to the LLPA group. Further studies are necessary to replicate our findings. 

Despite the limitations, the current study contains several findings of clinical relevance. On the one hand, HLPA may have a negative influence on the course of AN. For example, our data suggest that HLPA may mask the development of AN from patients and their social surroundings. This fact may cause a delay in searching for professional help and may prolong the time of illness without appropriate treatment. On the other hand, one should distinguish possibly different influences of HLPA on health status of AN patients during disease onset, maintenance, and recovery from AN. First, in our sample, HLPA did not have negative consequences on the recovery rate of adolescent AN patients. This observation surely results from the fact that the treatment was successful in diminishing the levels of PA in our sample. HLPA was, however, associated with higher body fat percentage and higher leptin levels in recovered patients (at the end of study and at follow-up) in comparison to the LLPA group. These observations may have negative as well as positive influences on long-term remission. It has been previously shown that high leptin levels may counteract therapeutic efforts by increasing PA and decreasing food intake [52–57]. However, in the current study, we did not observe an increase in PA at one-year follow-up in the group of patients with high plasma leptin levels. Furthermore, the correlation analysis has shown that the higher the fat% and leptin levels, the better the psychological outcome as measured using CPRS-S-A and EDI-2 questionnaires. This may suggest that although HLPA contribute to the development of AN and may counteract treatment effort, it may also have a positive side. Namely, HLPA patients who recover and manage to maintain low PA levels may have better treatment outcomes than LLPA patients. It is possible that previous HLPA result in higher fat% levels after refeeding, which may in turn result in higher leptin plasma levels in comparison to the LLPA group. This may have an unexpected positive influence on the psychological status of AN patients as leptin is known to diminish hypothalamic-pituitary-adrenal axis response to stress [58]. This hypothesis requires, however, further research as well as replication of current findings in a larger sample.

In summary, the data show that AN patients who are characterized by HLPA at the acute phase of the illness are characterized by longer duration of illness prior to admission to specialized treatment. High activity levels in adolescent AN patients do not hamper per se the progress in treatment. However, body composition recovery differs between the recovered LLPA and the recovered HLPA groups. Namely, the increase in fat% is higher for the recovered HLPA than for the recovered LLPA patients, even at one-year follow-up. Furthermore, these findings suggest that HLPA at initial assessment have long lasting consequences for body composition and consequently for levels of psychopathology at recovery.

## Supporting Information

Table S1 **Physical activity changes during treatment.** Results of repeated measures ANOVA on changes of PA (physical activity) during treatment. Significant *P*-values are marked in bold. ^*^ɛ=0.764.
(DOCX)

Table S2 **Results of the two-way ANOVA analyses.** Results of the two-way ANOVA on differences between the recovered and the non-recovered LLPA and HLPA AN patients. As = initial assessment, EoS = the end of study, F-up = follow-up. Significant *P*-values are marked in bold. ^*^ɛ=0.764.
(DOCX)

Table S3 **Correlations between biological and psychological variables at follow-up.** Spearman’s rho correlation coefficients for correlations between fat%, plasma leptin, and plasma ghrelin levels with psychological variables measured using the Eating Disorder Inventory (EDI-2) and the Comprehensive Psychopathological Rating Scale (CPRS-S-A). Interpers. distrust = interpersonal distrust; interoceptive aw.= interoceptive awareness. * *P*<.05, ** *P*<.01. Numbers in the brackets indicate the sample size in a given correlation analysis.
(DOCX)

## References

[1] Gull WW (1997) Anorexia nervosa (apepsia hysterica, anorexia hysterica). 1868. Obes Res 5: 498-502. doi:10.1002/j.1550-8528.1997.tb00677.x. PubMed: 9385628. 9385628

[2] Pearce JM (2004) Richard Morton: Origins of anorexia nervosa. Eur Neurol 52: 191-192. doi:10.1159/000082033. PubMed: 15539770. 15539770

[3] Hebebrand J, Exner C, Hebebrand K, Holtkamp C, Casper RC et al. (2003) Hyperactivity in patients with anorexia nervosa and in semistarved rats: evidence for a pivotal role of hypoleptinemia. Physiol Behav 79: 25-37. doi:10.1016/S0031-9384(03)00102-1. PubMed: 12818707. 12818707

[4] Davis C, Kennedy SH, Ravelski E, Dionne M (1994) The role of physical activity in the development and maintenance of eating disorders. Psychol Med 24: 957-967. doi:10.1017/S0033291700029044. PubMed: 7892363. 7892363

[5] Hubbard S, Gray JJ, Parker S (1998) Differences Among Women who Exercise for `Food Related' and `Non-food Related' Reasons. Eur Eat Disord Rev 6: 255-265. doi:10.1002/(SICI)1099-0968(199812)6:4.

[6] Mond JM, Hay PJ, Rodgers B, Owen C (2006) An update on the definition of "excessive exercise" in eating disorders research. Int J Eat Disord 39: 147-153. doi:10.1002/eat.20214. PubMed: 16231344. 16231344

[7] Davis C, Brewer H, Ratusny D (1993) Behavioral frequency and psychological commitment: necessary concepts in the study of excessive exercising. J Behav Med 16: 611-628. doi:10.1007/BF00844722. PubMed: 8126715. 8126715

[8] Alberti M, Galvani C, El Ghoch M, Capelli C, Lanza M et al. (2013) Assessment of Physical Activity in Anorexia Nervosa and Treatment Outcome. Med Sci Sports Exerc 45: 1643-1648. doi:10.1249/MSS.0b013e31828e8f07. PubMed: 23475165. 23475165

[9] Beumont PJ, Arthur B, Russell JD, Touyz SW (1994) Excessive physical activity in dieting disorder patients: proposals for a supervised exercise program. Int J Eat Disord 15: 21-36. doi:10.1002/1098-108X(199401)15:1. PubMed: 8124324. 8124324

[10] Casper RC, Jabine LN (1996) An eight-year follow-up: Outcome from adolescent compared to adult onset anorexia nervosa. J Youth Adolesc 25: 499-517. doi:10.1007/BF01537545.

[11] Strober M, Freeman R, Morrell W (1997) The long-term course of severe anorexia nervosa in adolescents: survival analysis of recovery, relapse, and outcome predictors over 10-15 years in a prospective study. Int J Eat Disord 22: 339-360. doi:10.1002/(SICI)1098-108X(199712)22:4. PubMed: 9356884. 9356884

[12] Shroff H, Reba L, Thornton LM, Tozzi F, Klump KL et al. (2006) Features associated with excessive exercise in women with eating disorders. Int J Eat Disord 39: 454-461. doi:10.1002/eat.20247. PubMed: 16637047. 16637047

[13] Bratland-Sanda S, Sundgot-Borgen J, Rø O, Rosenvinge JH, Hoffart A et al. (2010) "I'm not physically active - I only go for walks": physical activity in patients with longstanding eating disorders. Int J Eat Disord 43: 88-92. PubMed: 19728373. 19728373 10.1002/eat.20753

[14] Holtkamp K (2004) High serum leptin levels subsequent to weight gain predict renewed weight loss in patients with anorexia nervosa. Psychoneuroendocrinology 29: 791-797. doi:10.1016/S0306-4530(03)00143-4. PubMed: 15110928. 15110928

[15] Carrera O, Adan RA, Gutierrez E, Danner UN, Hoek HW et al. (2012) Hyperactivity in anorexia nervosa: warming up not just burning-off calories. PLOS ONE 7: e41851. doi:10.1371/journal.pone.0041851. PubMed: 22848634. 22848634 PMC3407098

[16] Kron L, Katz JL, Gorzynski G, Weiner H (1978) Hyperactivity in anorexia nervosa: a fundamental clinical feature. Compr Psychiatry 19: 433-440. doi:10.1016/0010-440X(78)90072-X. PubMed: 679677. 679677

[17] Epling W, Pierce W (1992) Solving the anorexia puzzle - a scientific approach. Toronto: Hogrefe & Huber Publishing House.

[18] Holtkamp K, Hebebrand J, Herpertz-Dahlmann B (2004) The contribution of anxiety and food restriction on physical activity levels in acute anorexia nervosa. Int J Eat Disord 36: 163-171. doi:10.1002/eat.20035. PubMed: 15282686. 15282686

[19] Inoko K, Nishizono-Maher A, Ishii K, Osawa M (2005) Effect of medical treatments on psychiatric symptoms in children with anorexia nervosa. Pediatr Int 47: 3: 326–8. PubMed: 15910460. 15910460 10.1111/j.1442-200x.2005.02072.x

[20] Hebebrand J, Muller TD, Holtkamp K, Herpertz-Dahlmann B (2007) The role of leptin in anorexia nervosa: clinical implications. Mol Psychiatry 12: 23-35. doi:10.1038/sj.mp.4001909. PubMed: 17060920. 17060920

[21] Köpp W, Blum WF, von Prittwitz S, Ziegler A, Lübbert H et al. (1997) Low leptin levels predict amenorrhea in underweight and eating disordered females. Mol Psychiatry 2: 335-340. doi:10.1038/sj.mp.4000287. PubMed: 9246675. 9246675

[22] Tolle V, Kadem M, Bluet-Pajot MT, Frere D, Foulon C et al. (2003) Balance in ghrelin and leptin plasma levels in anorexia nervosa patients and constitutionally thin women. J Clin Endocrinol Metab 88: 109-116. doi:10.1210/jc.2002-020645. PubMed: 12519838. 12519838

[23] von Prittwitz S, Blum WF, Ziegler A, Scharmann S, Remschmidt H et al. (1997) Restrained eating is associated with low leptin levels in underweight females. Mol Psychiatry 2: 420-422. doi:10.1038/sj.mp.4000300. PubMed: 9322239. 9322239

[24] Mayo-Smith W, Hayes CW, Biller BM, Klibanski A, Rosenthal H et al. (1989) Body fat distribution measured with CT: correlations in healthy subjects, patients with anorexia nervosa, and patients with Cushing syndrome. Radiology 170: 515-518. PubMed: 2911678. 2911678 10.1148/radiology.170.2.2911678

[25] Mayer LE, Klein DA, Black E, Attia E, Shen W et al. (2009) Adipose tissue distribution after weight restoration and weight maintenance in women with anorexia nervosa. Am J Clin Nutr 90: 1132-1137. doi:10.3945/ajcn.2009.27820. PubMed: 19793856.. 19793856 PMC2762154

[26] Mayer L, Walsh BT, Pierson RN Jr., Heymsfield SB, Gallagher D et al. (2005) Body fat redistribution after weight gain in women with anorexia nervosa. Am J Clin Nutr 81: 1286-1291. PubMed: 15941877. 15941877 10.1093/ajcn/81.6.1286

[27] Orphanidou CI, McCargar LJ, Birmingham CL, Belzberg AS (1997) Changes in body composition and fat distribution after short-term weight gain in patients with anorexia nervosa. Am J Clin Nutr 65: 1034-1041. PubMed: 9094890. 9094890 10.1093/ajcn/65.4.1034

[28] Iketani T, Kiriike N, Nagata T, Yamagami S (1999) Altered body fat distribution after recovery of weight in patients with anorexia nervosa. Int J Eat Disord 26: 275-282. doi: 275-282. 10.1002/(SICI)1098-108X(199911)26:3<275::AID-EAT4>3.0.CO;2-I [pii].. PubMed: 10441242. 10441242 10.1002/(sici)1098-108x(199911)26:3<275::aid-eat4>3.0.co;2-i

[29] Grinspoon S, Thomas L, Miller K, Pitts S, Herzog D et al. (2001) Changes in regional fat redistribution and the effects of estrogen during spontaneous weight gain in women with anorexia nervosa. Am J Clin Nutr 73: 865-869. PubMed: 11333838. 11333838 10.1093/ajcn/73.5.865

[30] Mathiak K, Gowin W, Hebebrand J, Ziegler A, Blum WF et al. (1999) Serum leptin levels, body fat deposition, and weight in females with anorexia or bulimia nervosa. Horm Metab Res 31: 274-277. doi:10.1055/s-2007-978732. 10333084

[31] Verhagen LA, Luijendijk MC, Hillebrand JJ, Adan RA (2009) Dopamine antagonism inhibits anorectic behavior in an animal model for anorexia nervosa. Eur Neuropsychopharmacol 19: 153-160. doi:10.1016/j.euroneuro.2008.09.005. PubMed: 18977121. 977X(08)00252-6 18977121

[32] Verhagen LA, Luijendijk MC, Adan RA (2011) Leptin reduces hyperactivity in an animal model for anorexia nervosa via the ventral tegmental area. Eur Neuropsychopharmacol 21: 274-281. 10.1016/S0924-977X(11)70429-1 PubMed: 21190812 977X(10)00244-0 .. 21190812

[33] Hillebrand JJ, Koeners MP, de Rijke CE, Kas MJ, Adan RA (2005) Leptin Treatment in Activity-Based Anorexia. Biol Psychiatry 58: 165-171. doi:10.1016/j.biopsych.2005.03.011. PubMed: 16038687. 16038687

[34] Holtkamp K, Herpertz-Dahlmann B, Mika C, Heer M, Heussen N et al. (2003) Elevated physical activity and low leptin levels co-occur in patients with anorexia nervosa. J Clin Endocrinol Metab 88: 5169-5174. doi:10.1210/jc.2003-030569. PubMed: 14602745. 14602745

[35] Exner C, Hebebrand J, Remschmidt H, Wewetzer C, Ziegler A et al. (2000) Leptin suppresses semi-starvation induced hyperactivity in rats: implications for anorexia nervosa. Mol Psychiatry 5: 476-481. doi:10.1038/sj.mp.4000771. PubMed: 11032380. 11032380

[36] Holtkamp K, Hebebrand J, Mika C, Grzella I, Heer M et al. (2003) The effect of therapeutically induced weight gain on plasma leptin levels in patients with anorexia nervosa. J Psychiatr Res 37: 165-169. doi:10.1016/S0022-3956(02)00100-0. PubMed: 12842170. S0022395602001000 . PII. 12842170

[37] Hebebrand J, Blum WF, Barth N, Coners H, Englaro P et al. (1997) Leptin levels in patients with anorexia nervosa are reduced in the acute stage and elevated upon short-term weight restoration. Mol Psychiatry 2: 330-334. doi:10.1038/sj.mp.4000282. PubMed: 9246674. 9246674

[38] Wabitsch M, Ballauff A, Holl R, Blum WF, Heinze E et al. (2001) Serum leptin, gonadotropin, and testosterone concentrations in male patients with anorexia nervosa during weight gain. J Clin Endocrinol Metab 86: 2982-2988. doi:10.1210/jc.86.7.2982. PubMed: 11443155. 11443155

[39] Popovic V, Djurovic M, Cetkovic A, Vojvodic D, Pekic S et al. (2004) Inhibin B: a potential marker of gonadal activity in patients with anorexia nervosa during weight recovery. J Clin Endocrinol Metab 89: 1838-1843. doi:10.1210/jc.2003-031326. PubMed: 15070953. 15070953

[40] Djurovic M, Pekic S, Petakov M, Damjanovic S, Doknic M et al. (2004) Gonadotropin response to clomiphene and plasma leptin levels in weight recovered but amenorrhoeic patients with anorexia nervosa. J Endocrinol Invest 27: 523-527. PubMed: 15717648. 15717648 10.1007/BF03347473

[41] Tolle V, Kadem M, Bluet-Pajot MT, Frere D, Foulon C et al. (2003) Balance in ghrelin and leptin plasma levels in anorexia nervosa patients and constitutionally thin women. J Clin Endocrinol Metab 88: 109-116. doi:10.1210/jc.2002-020645. PubMed: 12519838. 12519838

[42] Soriano-Guillén L, Barrios V, Campos-Barros A, Argente J. (2004) Ghrelin levels in obesity and anorexia nervosa: effect of weight reduction or recuperation J Pediatr, 144: 36–42 10.1016/j.jpeds.2003.10.036 14722516

[43] Inui A (2001) Ghrelin: an orexigenic and somatotrophic signal from the stomach. Nat Rev Neurosci 2: 551-560. doi:10.1038/35086018. PubMed: 11483998. 35086018 . PII. 11483998

[44] Otto B, Cuntz U, Fruehauf E, Wawarta R, Folwaczny C et al. (2001) Weight gain decreases elevated plasma ghrelin concentrations of patients with anorexia nervosa. Eur J Endocrinol 145: 669-673. doi:10.1530/eje.0.1450669. PubMed: 11720888. 145669 . PII. 11720888

[45] Probst M, Knapen J, Poot G, Vancampfort D (2010) Psychomotor therapy and psychiatry: what's in a name? Open Complement Med J 2: 105-113. doi:10.2174/1876391X010020010105.

[46] Lee S, Chan YY, Hsu LK (2003) The intermediate-term outcome of Chinese patients with anorexia nervosa in Hong Kong. Am J Psychiatry 160: 967-972. doi:10.1176/appi.ajp.160.5.967. PubMed: 12727702. 12727702

[47] Puyau MR, Adolph AL, Vohra FA, Butte NF (2002) Validation and calibration of physical activity monitors in children. Obes Res 10: 150-157. doi:10.1038/oby.2002.24. PubMed: 11886937. 11886937

[48] Davis C (1995) Obsessive compulsiveness and physical activity in anorexia nervosa and high-level exercising. J Psychosom Res 39: 967-976. doi:10.1016/0022-3999(95)00064-X. PubMed: 8926606. 8926606

[49] Thome JL, Espelage DL (2007) Obligatory exercise and eating pathology in college females: replication and development of a structural model. Eat Behav 8: 334-349. doi:10.1016/j.eatbeh.2006.11.009. PubMed: 17606231. 0153(06)00087-0 . 17606231

[50] Carter JC, Blackmore E, Sutandar-Pinnock K, Woodside DB (2004) Relapse in anorexia nervosa: a survival analysis. Psychol Med 34: 671-679. doi:10.1017/S0033291703001168. PubMed: 15099421. 15099421

[51] Gelegen C, Collier DA, Campbell IC, Oppelaar H, van den Heuvel J et al. (2007) Difference in susceptibility to activity-based anorexia in two inbred strains of mice. Eur Neuropsychopharmacol 17: 199-205. doi:10.1016/S0924-977X(07)70233-X. PubMed: 16735105. 16735105

[52] Hwa JJ, Fawzi AB, Graziano MP, Ghibaudi L, Williams P et al. (1997) Leptin increases energy expenditure and selectively promotes fat metabolism in ob/ob mice. Am J Physiol 272: R1204-R1209. PubMed: 9140021. 9140021 10.1152/ajpregu.1997.272.4.R1204

[53] Farooqi IS, Matarese G, Lord GM, Keogh JM, Lawrence E et al. (2002) Beneficial effects of leptin on obesity, T cell hyporesponsiveness, and neuroendocrine/metabolic dysfunction of human congenital leptin deficiency. J Clin Invest 110: 1093-1103. doi:10.1172/JCI15693. PubMed: 12393845. 12393845 PMC150795

[54] Heymsfield SB, Greenberg AS, Fujioka K, Dixon RM, Kushner R et al. (1999) Recombinant leptin for weight loss in obese and lean adults: a randomized, controlled, dose-escalation trial. JAMA 282: 1568-1575. doi:10.1001/jama.282.16.1568. PubMed: 10546697. jpc90045 . PII. 10546697

[55] Scarpace PJ, Matheny M, Pollock BH, Tümer N (1997) Leptin increases uncoupling protein expression and energy expenditure. Am J Physiol 273: E226-E230. PubMed: 9252501. 9252501 10.1152/ajpendo.1997.273.1.E226

[56] van Dijk G (2001) The role of leptin in the regulation of energy balance and adiposity. J Neuroendocrinol 13: 913-921. PubMed: 11679060. 11679060 10.1046/j.1365-2826.2001.00707.x

[57] van Elburg AA, Kas MJ, Hillebrand JJ, Eijkemans RJ, van Engeland H (2007) The impact of hyperactivity and leptin on recovery from anorexia nervosa. J Neural Transm 114: 1233-1237. doi:10.1007/s00702-007-0740-6. PubMed: 17530161. 17530161 PMC2798977

[58] Malendowicz LK, Rucinski M, Belloni AS, Ziolkowska A, Nussdorfer GG (2007) Leptin and the regulation of the hypothalamic-pituitary-adrenal axis. Int Rev Cytol 263: 63-102. doi:10.1016/S0074-7696(07)63002-2. PubMed: 17725965. 7696(07)63002-2 . 17725965

