# Supplementary material for: Longitudinal Changes in the Physical Activity of Adolescents with Anorexia Nervosa and Their Influence on Body Composition and Leptin Serum Levels after Recovery
Source: PLoS One. 2013 Oct 21;8(10):e78251. doi: 10.1371/journal.pone.0078251 (PMC3804495; doi:10.1371/journal.pone.0078251)
Supplement: Table S1 — Physical activity changes during treatment. Results of repeated measures ANOVA on changes of PA (physical activity) during treatment. Significant P-values are marked in bold. *ɛ=0.764. (DOCX) [file pone.0078251.s001.docx]

Table S1. Physical activity changes during treatment.

| Repeated measures ANOVA | | | | | | | | | |
| --- | --- | --- | --- | --- | --- | --- | --- | --- | --- |
|  | PA classification | | | Time | | | Interaction | | |
|  | F (df) | *P* < | η^2^partial | F (df) | *P* < | η^2^partial | F (df) | *P* < | η^2^partial |
| PA | 4.392 (1,35) | **0.05** | 0.111 | 0.002 (1.53,53.47)^*^ | 1.00 | <0.001 | 11.758 (1.53) | **0.001** | 0.273 |

Results of repeated measures ANOVA on changes of PA (physical activity) during treatment. Significant *P*-values are marked in bold. ^*^ɛ=0.764.
