# Supplementary material for: Longitudinal Changes in the Physical Activity of Adolescents with Anorexia Nervosa and Their Influence on Body Composition and Leptin Serum Levels after Recovery
Source: PLoS One. 2013 Oct 21;8(10):e78251. doi: 10.1371/journal.pone.0078251 (PMC3804495; doi:10.1371/journal.pone.0078251)
Supplement: Table S2 — Results of the two-way ANOVA analyses. Results of the two-way ANOVA on differences between the recovered and the non-recovered LLPA and HLPA AN patients. As = initial assessment, EoS = the end of study, F-up = follow-up. Significant P-values are marked in bold. *ɛ=0.764. (DOCX) [file pone.0078251.s002.docx]

Table S2. Results of the two-way ANOVA analyses.

| Two-way ANOVA | | | | | | | | | |
| --- | --- | --- | --- | --- | --- | --- | --- | --- | --- |
|  | PA classification | | | Recovery status | | | Interaction | | |
|  | F (df) | *P* < | η^2^partial | F (df) | *P* < | η^2^partial | F (df) | *P* < | η^2^partial |
| PA |  |  |  |  |  |  |  |  |  |
| As | 74.764 (1,33) | **0.001** | 0.69 | 12.432 (1,33) | **0.002** | 0.27 | 9.972(1,33) | **0.004** | 0.23 |
| EoS | 0.146 (1,33) | 0.71 | 0.004 | 0.163 (1,33) | 0.69 | 0.005 | 1.241 (1,33) | 0.27 | 0.04 |
| F-up | 0.032 (1,33) | 0.86 | 0.001 | 0.382 (1,33) | 0.54 | 0.006 | 1.922 (1,33) | 0.18 | 0.03 |
| Fat% |  |  |  |  |  |  |  |  |  |
| As | 0.949 (1,31) | 0.34 | 0.03 | 0.040 (1,31) | 0.84 | 0.001 | 0.272 (1,31) | 0.61 | 0.01 |
| EoS | 0.705 (1,32) | 0.41 | 0.02 | 7.005 (1,32) | **0.05** | 0.18 | 0.328 (1,32) | 0.57 | 0.01 |
| F-up | 0.968 (1,21) | 0.34 | 0.07 | 17.602 (1,21) | **0.001** | 0.23 | 5.404 (1,21) | **0.03** | 0.11 |
| Leptin |  |  |  |  |  |  |  |  |  |
| EoS | 1.432 (1,29) | 0.24 | 0.05 | 14.295 (1,29) | **0.001** | 0.33 | 4.001 (1,29) | 0.06 | 0.12 |
| F-up | 1.450 (1,17) | 0.25 | 0.17 | 20.087 (1,17) | **0.001** | 0.32 | 1.297 (1,17) | 0.27 | 0.32 |
| Ghrelin |  |  |  |  |  |  |  |  |  |
| EoS | 0.199 (1,29) | 0.66 | 0.007 | 1.679 (1,29) | 0.206 | 0.06 | 0.078 (1,29) | 0.78 | 0.003 |
| F-up | 0.334 (1,17) | 0.57 | 0.02 | 0.004 (1,17) | 0.95 | 0.03 | 0.043 (1,17) | 0.84 | 0.06 |

Results of the two-way ANOVA on differences between the recovered and the non-recovered LLPA and HLPA AN patients. As = initial assessment, EoS = the end of study, F-up = follow-up. Significant *P*-values are marked in bold. ^*^ɛ=0.764.
