# Supplementary material for: Longitudinal Changes in the Physical Activity of Adolescents with Anorexia Nervosa and Their Influence on Body Composition and Leptin Serum Levels after Recovery
Source: PLoS One. 2013 Oct 21;8(10):e78251. doi: 10.1371/journal.pone.0078251 (PMC3804495; doi:10.1371/journal.pone.0078251)
Supplement: Table S3 — Correlations between biological and psychological variables at follow-up. Spearman’s rho correlation coefficients for correlations between fat%, plasma leptin, and plasma ghrelin levels with psychological variables measured using the Eating Disorder Inventory (EDI-2) and the Comprehensive Psychopathological Rating Scale (CPRS-S-A). Interpers. distrust = interpersonal distrust; interoceptive aw.= interoceptive awareness. * P<.05, ** P<.01. Numbers in the brackets indicate the sample size in a given correlation analysis. (DOCX) [file pone.0078251.s003.docx]

Table S3. Correlations between biological and psychological variables at follow-up.

|  | Fat% | Leptin | Ghrelin |
| --- | --- | --- | --- |
| CPRS-S-A |  |  |  |
| anxiety | -0.414* (25) | -0.568** (21) | 0.399 (21) |
| obsessions | -0.480* (25) | -0.624** (21) | 0.167 (21) |
| depression | -0.496* (25) | -0.612** (21) | 0.345 (21) |
| EDI-2 |  |  |  |
| drive for thinness | -0.084 (25) | -0.192 (21) | 0.300 (21) |
| body dissatisfaction | 0.124 (25) | -0.415 (21) | 0.091 (21) |
| bulimia scale | -0.252 (25) | -0.227 (21) | 0.299 (21) |
| ineffectiveness | -0.542** (25) | -0.639** (21) | 0.439* (21) |
| perfectionism | -0.269 (25) | -0.245 (21) | 0.246 (21) |
| interpers. distrust | -0.559** (25) | -0.695** (21) | 0.445* (21) |
| interoceptive aw. | -0.323 (25) | -0.651** (21) | 0.319 (21) |
| maturity fears | -0.540** (25) | -0.674** (21) | 0.383 (21) |
| ascetism | 0.096 (25) | -0.425 (21) | 0.359 (21) |
| impulse regulation | 0.316 (25) | -0.613** (21) | 0.105 (21) |
| social insecurity | -0.625** (25) | -0.733** (21) | 0.254 (21) |

Spearman’s rho correlation coefficients for correlations between fat%, plasma leptin, and plasma ghrelin levels with psychological variables measured using the Eating Disorder Inventory (EDI-2) and the Comprehensive Psychopathological Rating Scale (CPRS-S-A). Interpers. distrust = interpersonal distrust; interoceptive aw.= interoceptive awareness. * *P*<.05, ** *P*<.01. Numbers in the brackets indicate the sample size in a given correlation analysis.
